# Adverse reactions to food in patients with mastocytosis

**DOI:** 10.1186/2045-7022-1-S1-P35

**Published:** 2011-08-12

**Authors:** Valentina Pucino, Diomira Magliacane, Angelica Petraroli, Massimo Triggiani

**Affiliations:** 1 Division of Clinical Immunology and Allergy, University of Naples Federico II, Naples, Italy

## Background

Mastocytosis is a disease characterized by abnormal growth and accumulation of mast cells (MC) in different tissues. The disease can be classified as cutaneous (CM), when MC infiltrate is limited to the skin, and systemic mastocytosis (SM) when MC proliferate in other organs such as the bone marrow, gastrointestinal tract, lung, liver, spleen or lymph-nodes. Clinical symptoms of CM and SM are related to the release of MC-derived mediators and/or to tissue infiltration by MC. The increased number of MC in various organs, including the gastrointestinal tract, raises the question whether patients with mastocytosis may have a high frequency of adverse reaction to food (ARF).

## Methods

We studied 126 patients (mean age 33 years; range 1-80 years) with mastocytosis, of which 34 (27%) were < 18 years old and 92 (73%) were > 18 years old. Mastocytosis was diagnosed according to WHO criteria (Valent et al. Leuk. Res. 25:603-25, 2001). Medical history of adverse reactions to food was obtained from all patients. Those who reported suspected reactions were further evaluated by skin prick test and serum specific IgE assay.

## Results

A positive history for ARF was detected in 12/34 (34%) patients < 18 years old and in 5/92 (5%) patients > 18 years old. Clinical manifestations of ARF were urticaria/angioedema (13 patients), asthma (2 patient) and anaphylaxis (2 patients). Eliciting food included peanuts (5 patients), nuts (3 patients), eggs (1 patient), tomatos (2 patients), peaches (2 patients) or shellfish (4 patients). A positive skin prick test and/or elevated levels of serum specific IgE for relevant food allergens were found only in 5 patients (4 < 18 years old).

## Conclusions

These results indicate that ARF are more frequent in pediatric than in adult patients with mastocytosis. The prevalence of ARF in adults with mastocytosis is not significantly different from that in the general population. Most of the adverse reactions to food in patients with mastocytosis are not associated with positive skin prick test or elevated serum specific IgE. These results suggest that mechanisms other than IgE-mediated may be involved in ARF in patients with mastocytosis.

